# Supplementary material for: Optimizing Irradiation Geometry in LED-Based Photoacoustic Imaging with 3D Printed Flexible and Modular Light Delivery System
Source: Sensors (Basel). 2020 Jul 6;20(13):3789. doi: 10.3390/s20133789 (PMC7374354; doi:10.3390/s20133789)
Supplement: Supplementary file 1 [file sensors-20-03789-s001.pdf]

sensors

Supplementary material

Optimizing Irradiation Geometry in LED-Based Photoacoustic Imaging with 3D Printed Flexible and Modular Light Delivery System

Maju Kuriakose [1], Christopher D. Nguyen [1], Mithun Kuniyil Ajith Singh [2], and Srivalleesha Mallidi [1,*]

[1] Department of Biomedical Engineering, Tufts University, Medford, MA 02155, USA; maju.kuriakose@tufts.edu (M.K.); Christopher.Nguyen@tufts.edu (C.D.N.)
[2] Cyberdyne Inc., Cambridge Innovation Center, Rotterdam, The Netherlands; mithun_ajith@cyberdyne.jp
* Correspondence: Srivalleesha.Mallidi@tufts.edu
Received: 21 May 2020; Accepted: 29 June 2020; Published: 6 July 2020

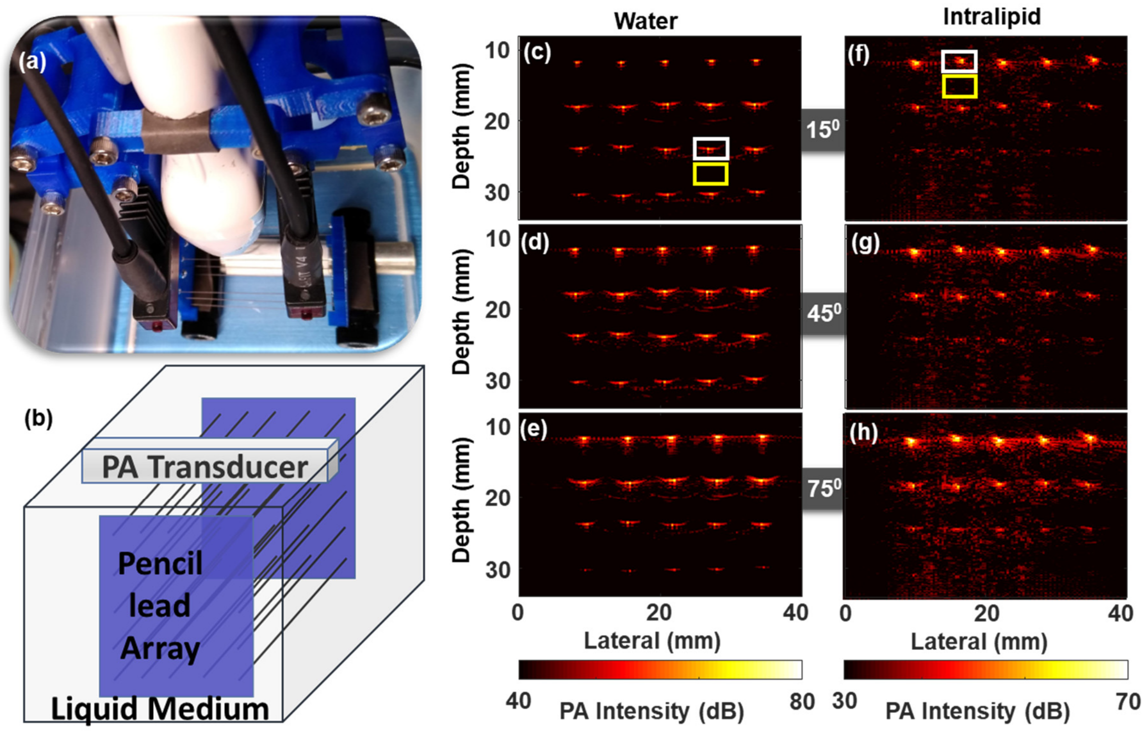

Figure S1: (a) A photograph of Pencil lead array placed in water bath in the presence of PA transducer on top; (b) Schematic of the pencil lead array; (c - h) PA image acquired at representative angles 15⁰ (c & f), 45⁰ (d & g), and 75⁰ (e & h) in water (c-e) and 1% intralipid (f-h); Differ to figure 2 in the main article, where the reconstructed images are shown in a dynamic range of 30 dB, these images are shown in 40 dB to show the presence of background.

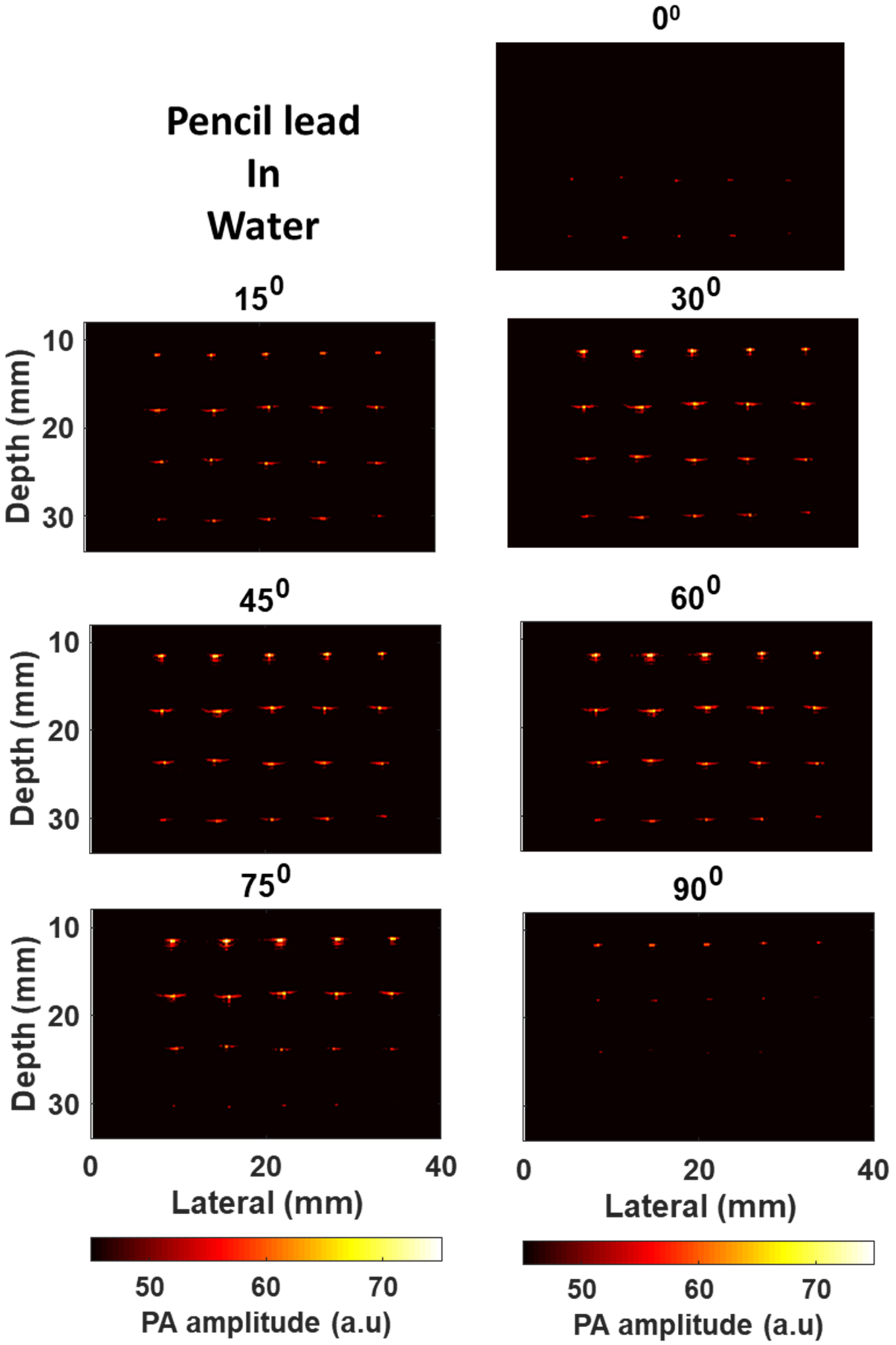

Figure S2: Pencil lead in water at different angles of illumination mentioned as in each image title. PA intensity values are given in the bottom color bar.

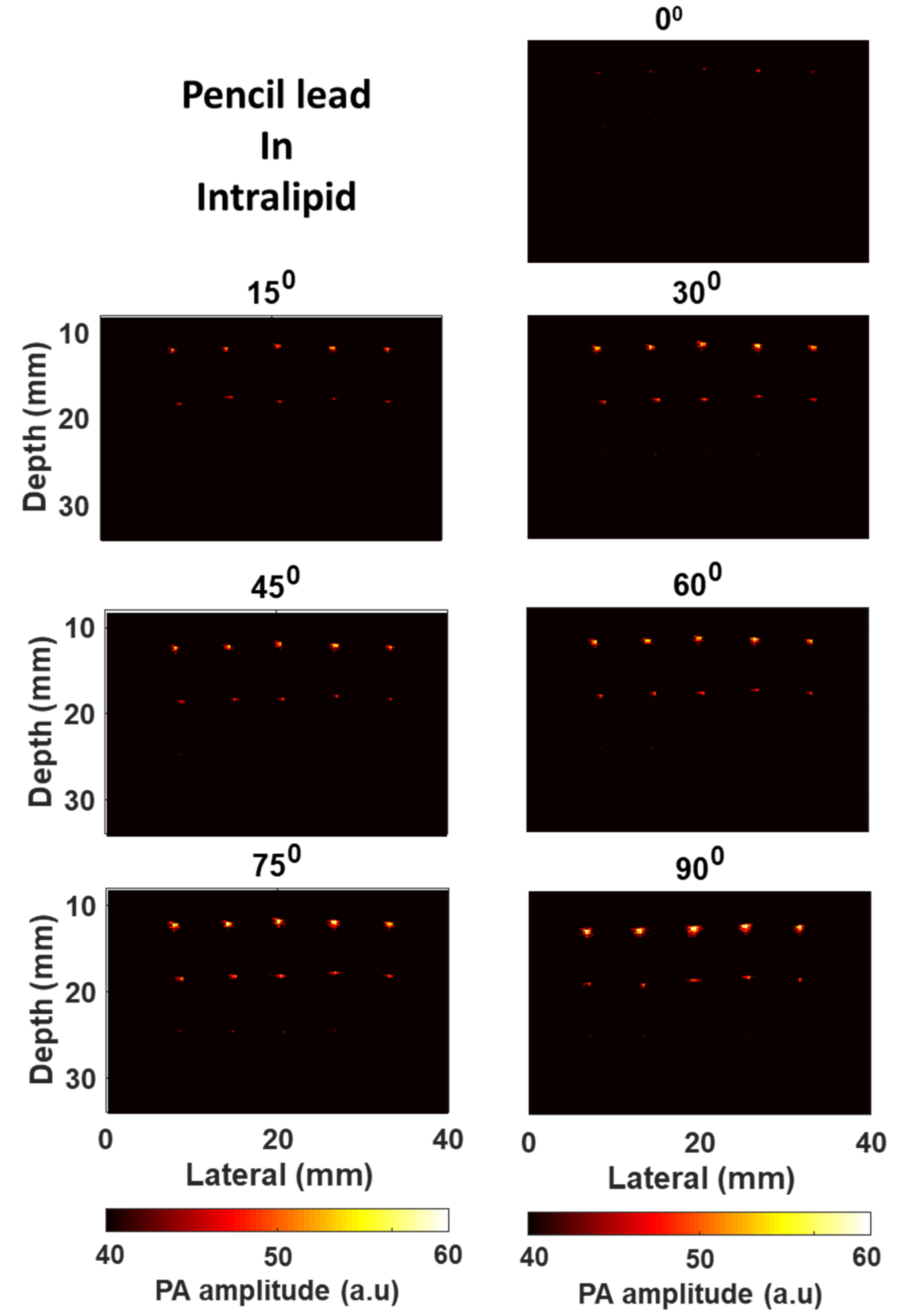

Figure S3: Pencil lead in intralipid at different angles of illumination mentioned as in each image title. PA intensity values are given in the bottom color bar.
